# Acute myocardial injury secondary to severe acute liver failure: A retrospective analysis supported by animal data

**DOI:** 10.1371/journal.pone.0256790

**Published:** 2021-08-30

**Authors:** Moritz Uhlig, Marc Hein, Moriz A. Habigt, René H. Tolba, Till Braunschweig, Marius J. Helmedag, Uwe Klinge, Alexander Koch, Christian Trautwein, Mare Mechelinck

**Affiliations:** 1 Department of Anesthesiology, Faculty of Medicine, RWTH Aachen University, Aachen, Germany; 2 Institute for Laboratory Animal Science and Experimental Surgery, Faculty of Medicine, RWTH Aachen University, Aachen, Germany; 3 Department of Pathology, Faculty of Medicine, RWTH Aachen University, Aachen, Germany; 4 Department of General, Visceral and Transplantation Surgery, Faculty of Medicine, RWTH Aachen University, Aachen, Germany; 5 Department of Gastroenterology, Faculty of Medicine, RWTH Aachen University, Aachen, Germany; Klinikum Region Hannover GmbH, GERMANY

## Abstract

To investigate whether acute liver failure (ALF) leads to secondary acute myocardial injury, 100 ALF patients that were retrospectively identified in a single center based on ICD 10 codes and 8 rats from an experimental study that died early after bile duct ligation (BDL) were examined. Creatine kinase (CK), creatine kinase-MB isoenzyme (CKMB) and cardiac troponin-I (cTnI) were analyzed as markers of myocardial injury. For histological analysis, *hematoxylin-eosin* (HE), *elastic Van Gieson* (EVG), CD41 and myeloperoxidase were used to stain rat hearts. Major adverse cardiac events (MACEs) were a critical factor for mortality (p = 0.037) in human ALF. Deceased patients exhibited higher levels of CKMB than survivors (p = 0.023). CKMB was a predictor of mortality in ALF (p = 0.013). Animals that died early after BDL exhibited increased cTnI, CKMB, tumor necrosis factor α (TNFα) and interleukin-6 (IL-6) levels compared to controls (cTnI: p = 0.011, CKMB: p = 0.008, TNFα: p = 0.003, IL-6: p = 0.006). These animals showed perivascular lesions and wavy fibers, microthrombi and neutrophilic infiltration in the heart. MACEs are decisive for mortality in human ALF, and elevated CKMB values indicate that this might be due to structural myocardial damage. Accordingly, CKMB was found to have predictive value for mortality in ALF. The results are substantiated by data from a rat BDL model demonstrating diffuse myocardial injury.

## 1 Introduction

Acute liver failure (ALF), with an approximate annual incidence of 5.5 cases per million inhabitants, is a rather rare disease, but with a mortality rate of 40%, it is still life-threatening [1]. It is the result of severe hepatocellular injury due to a variety of triggers, such as acetaminophen intoxication or viral hepatitis, to name just two of the most prevalent examples. A widely accepted definition of ALF consists of the combination of a coagulation abnormality with an international normalized ratio (INR) > 1.5, encephalopathy and the absence of pre-existing cirrhotic diseases [2, 3]. Since patients with ALF are prone to deteriorate quickly, an early transfer to an intensive care unit in a transplant center is mandatory [4]. However, despite the huge progress that has been made in the field of intensive care medicine over the last decade, the data available on ALF therapy are still poor. One reason for this is probably the rarity of ALF, which makes sufficiently powered clinical trials difficult. To date, the high mortality rate of ALF has been attributed to cerebral edema, bleeding and sepsis but rarely to cardiac complications [5].

### 1.1 Clinical trials

To close this knowledge gap, the *Acute Liver Failure Study Group* was founded in 1997 by the *National Institutes of Health* and has enrolled over 3000 patients to date [6]. Parekh *et al*. investigated 187 cases (the database contained 1038 patients by then) consecutively enrolled from May 1989 to August 2000. They discovered that 74% of their cohort had elevated cardiac troponin I (cTnI) values. Moreover, the cTnI levels correlated with increased mortality. Therefore, the authors concluded that subclinical myocardial injury might be a critical element of ALF [7].

On the other hand, Audimoolam *et al*. confirmed the elevated cTnI values in ALF patients but failed to show that elevated cTnI values correlate with cardiac dysfunction. Hence, they interpreted the increased cTnI values as an expression of metabolic stress [8].

Further studies have suggested that elevated troponin values occur in critically ill patients without initial cardiac disease and have a negative impact on prognosis, albeit not as an independent prognostic factor [9, 10]. Moreover, in a trial from 1976, more than 90% of ALF patients examined showed cardiac abnormalities, 25% of whom suffered sudden cardiac death [11].

### 1.2 Animal experiments

The clinical evidence is supplemented by results from experimental animal models: porcine ALF models investigating ALF induction by either surgical devascularization of the liver or hepatic ischemia/reperfusion injury showed significantly increased cTnI values compared to baseline, which were not observed in sham-operated controls [12, 13]. These results were paralleled by histological findings showing myocardial cell necrosis and edema. Furthermore, in a murine cholestasis model induced by bile duct ligation (BDL), a marked increase in oxidative stress and apoptosis in myocardial cells was reported [14].

Taking all these observations together, there are strong indications for the importance of a liver-heart axis in the clinical course of ALF patients.

### 1.3 Observations, analyses and working hypothesis

In line with the literature described above, we observed an unexpectedly high mortality rate of 20% (8/40) in rats within the first days after BDL, which appeared to be partially related to acute myocardial injury. Therefore, we hypothesized that ALF may lead to secondary myocardial injury, that may have a direct impact on mortality.

To further test this hypothesis, a retrospective analysis of 100 ALF patients was performed to investigate the relationship between cardiac in-hospital complications, myocardial injury markers and mortality in ALF. Thus, this paper combines animal experimental observations with retrospective analysis of patient data.

## 2 Materials and methods

### 2.1 Animals and animal model

The original animal experiment was planned to investigate the effect of liver cirrhosis on vascular remodeling in rats. Thus, the actual study describes unexpected cardiac complications that were not the focus of the primary investigation.

All experiments were performed in male *Sprague-Dawley* rats (*Charles River Laboratories International*, *Inc*., Sulzfeld, Germany) with an average weight of 489.8 g±29.07 g. All rats were housed in an environmentally controlled room with a 12-hour light/dark cycle in which food and water were freely available at all times. Prior to the experiments, all animals were given 7 days to acclimatize; during this time, no interventions were performed. All experimental procedures were within the German Animal Welfare Act (§ 8 Abs. 1, *Tierschutzgesetz* [15]) and were approved by the governmental animal care and use office (No 84–02.04.2016. A391, *Landesamt für Natur*, *Umwelt und Verbraucherschutz Nordrhein-Westfalen*, Recklinghausen, Germany).

Cirrhosis was induced by ligation of the common bile duct (CBD) as described by Tag *et al*. [16]. The rats were anesthetized using isoflurane (2 vol%), and perioperative analgesia was ensured by subcutaneous administration of buprenorphine (0.01–0.03 mg/kg body weight, *Temgesic*, *Essex Pharma Gmbh—Msd Sharp & Dohme Gmbh*, Haar, Germany) 30 minutes prior to intervention. The abdominal cavity was opened via median laparotomy with subsequent preparation of the CBD. It was ligated twice with a silk thread 5/0 (18020–50, *Fine Science Tools*, Vancouver, Canada) and transected between the ligations. Sham-operated animals served as controls, and the same procedure was followed, except for the ligatures and transection of the CBD.

The above-described ligature of the CBD or the sham operation was performed on day 1 of the experiment. Within the initially planned experiment, balloon dilatation of the left carotid artery was performed 4 weeks after BDL. Before every intervention, transthoracic echocardiography (TTE) was performed as described below. Depending on the assigned experimental group, the animals received a third TTE and blood sampling 3, 7, 14 or 28 days after balloon dilatation. The animals were then euthanized under deep anesthesia by exsanguination in combination with heart removal. The heart and liver were preserved at each time point. For the current investigation, only serum and tissue samples from sham animals from day 3 after balloon dilatation (31 days after the first surgery) served as controls, as this was the earliest scheduled endpoint.

Throughout the study, the animals were visited at least daily, and their general condition was assessed using a semiquantitative score. The score considered body weight, general condition, spontaneous behavior and behavior-specific criteria (5–9 points = low stress, 10–19 points = medium stress, and ≥ 20 points = high stress). If the animals presented more than 20 points or 10–19 points longer than 72 h, the animal welfare officers were immediately consulted, and where appropriate, the animals were euthanized without delay. In that case, the animals were processed according to the same protocol as planned for the regular endpoint. If the animals died prematurely without euthanasia and therefore did not necessarily die under supervision, the best possible sampling (preservation of heart and liver) was performed. Quasi-randomization was used to assign animals to the respective intervention groups. Because the animals were visually distinguishable after the intervention, the advantage of true randomization would have been eliminated in any case. The primary outcome measure was premature death after BDL. The groups were as follows: control n = 9 animals, BDL surviving n = 6 animals, BDL dead n = 8 animals. A total of 23 animals was reported in this study.

### 2.2 Control of the successful induction of the ALF

To ensure that BDL caused significant injury to the liver, ALT, AST and total bilirubin were measured as part of the biochemical analysis. In addition, a histological work-up of the liver tissue was performed. Liver sections were fixed in 4% buffered formalin (*ROTI Histofix 4%*, *Carl Roth GmbH + Co*. *KG*, Karlsruhe, Germany) for one week, subsequently embedded in paraffin and cut into slices of 3 μm thickness. The prepared slides were then dewaxed and rehydrated using a standard xylol and a descending alcohol series. Histological examination of liver tissue was performed by means of *hematoxylin & eosin staining* (*Merck KGaA*, Darmstadt, Germany). HE staining was conducted according to a standard protocol.

### 2.3 Blood analysis to evaluate myocardial impairment in rats

For biochemical analysis, the total amount of blood that could be preserved was taken immediately postmortem. As a marker of myocardial injury, cTnI was measured using a commercially available ELISA Kit (Rat Cardiac Troponin-I ELISA, CTNI-2-HS; *Life Diagnostics*, *Inc*., West Chester, Pa., USA). For the assessment of inflammatory processes, interleukin-6 (IL-6) and tumor necrosis factor α (TNFα) were measured using an *R&D systems* ELISA Kit (Rat IL-6 Quantikine ELISA Kit, R6000B; Rat TNF-alpha Quantikine ELISA Kit, RTA00; *R&D Systems*, *Inc*. Minneapolis, MN, USA). All assays were performed according to the respective manufacturer’s protocols.

Creatine kinase (CK) and creatine kinase-MB isoenzyme (CKMB) were determined by the laboratory for hematology at the *Institute for Laboratory Animal Science and Experimental Surgery*, *RWTH Aachen University*, *Faculty of Medicine*, *Aachen*, *Germany*.

### 2.4 Histological evaluation of myocardial impairment in rats

Midpapillary sections of rat hearts were fixed in 4% buffered formalin (*ROTI Histofix 4%*, *Carl Roth GmbH + Co*. *KG*, Karlsruhe, Germany) for one week, subsequently embedded in paraffin and cut into slices of 3 μm thickness. The prepared slides were then dewaxed and rehydrated using a standard xylol and a descending alcohol series. Histological examination of the myocardial tissue was performed by means of *hematoxylin & eosin staining* (*Merck KGaA*, Darmstadt, Germany) and *elastic Van Gieson staining (Merck KGaA*, Darmstadt, Germany; *Chroma*, *Waldeck GmbH & Co*. *KG*, Muenster, Germany). All stains were conducted according to a standard protocol.

For immunohistological staining, the *ZytoChem-Plus AP Polymer-Kit* (*Zytomed Systems GmbH*, Berlin, Germany) was used according to the manufacturer’s protocol. To detect microthrombi, cluster of differentiation 41 (CD41) antibody (ab 203189, *Abcam*, Berlin, Germany) and neutrophil myeloperoxidase (MPO) antibody (ab 208670, *Abcam*, Berlin, Germany) were used as primary antibodies. The evaluation of the stained slides was performed in collaboration with the *Department of Pathology*, *RWTH Aachen University*, *Faculty of Medicine*, *Aachen*, *Germany*.

### 2.5 Functional evaluation of myocardial impairment

#### 2.5.1 Monitoring (noninvasive blood pressure (NIBP), electrocardiography (ECG) and oxygen saturation (SpO2))

After induction of anesthesia, rats were placed in a supine position, and cardiovascular monitoring consisting of pulse oximetry for SpO2 measurement, ECG and noninvasive measurement of NIBP was established.

Pulse oximetry was placed at the rodent’s paw by means of a commercially available infrared system (*Radical-7*, *Masimo*, *Puchheim*, Germany). A 3-channel ECG according to Eindhoven/Goldberger was derived using needle electrodes and a differential amplifier (*BioAmp FE231*, *ADInstruments Ltd*., Oxford, UK). In addition, NIBP measurement was performed using a commercially available device (*IN125/R*, *ADInstruments Ltd*., Oxford, UK) consisting of an inflatable tail cuff system in combination with a pulse sensor.

All parameters were measured and recorded during the entire time of surgery and stored for further analysis using *Labchart* (*ADInstruments Ltd*., Oxford, UK).

#### 2.5.2 Echocardiography and strain analysis

After monitoring was established as described above, TTE was performed prior to surgery: a 10 MHz transducer (*GE 10S-RS*, *GE Healthcare*, Chicago, IL, USA) connected to a *Vivid I* (*GE Healthcare*, Chicago, IL, USA) was used. Echocardiographic examination was performed according to a fixed protocol: short axis (SAX) and SAX motion mode (M-mode) in the midpapillary plane, long axis (LAX), aorta and M-mode aorta, pulmonary artery and continuous wave (CW) Doppler of the pulmonary artery, apical four-chamber view (A4C) and CW Doppler of the mitral valve in the A4C. The target parameters of the examination were ejection fraction (EF), stroke volume (SV), strain and diastolic function. The analysis of the acquired data was performed via *EchoPAC software* (Version 201; *GE Healthcare*, Chicago, IL, USA). Echocardiographic values of the prematurely deceased animals were compared with their respective controls before BDL.

### 2.6 Clinical data

**Fig 1:**
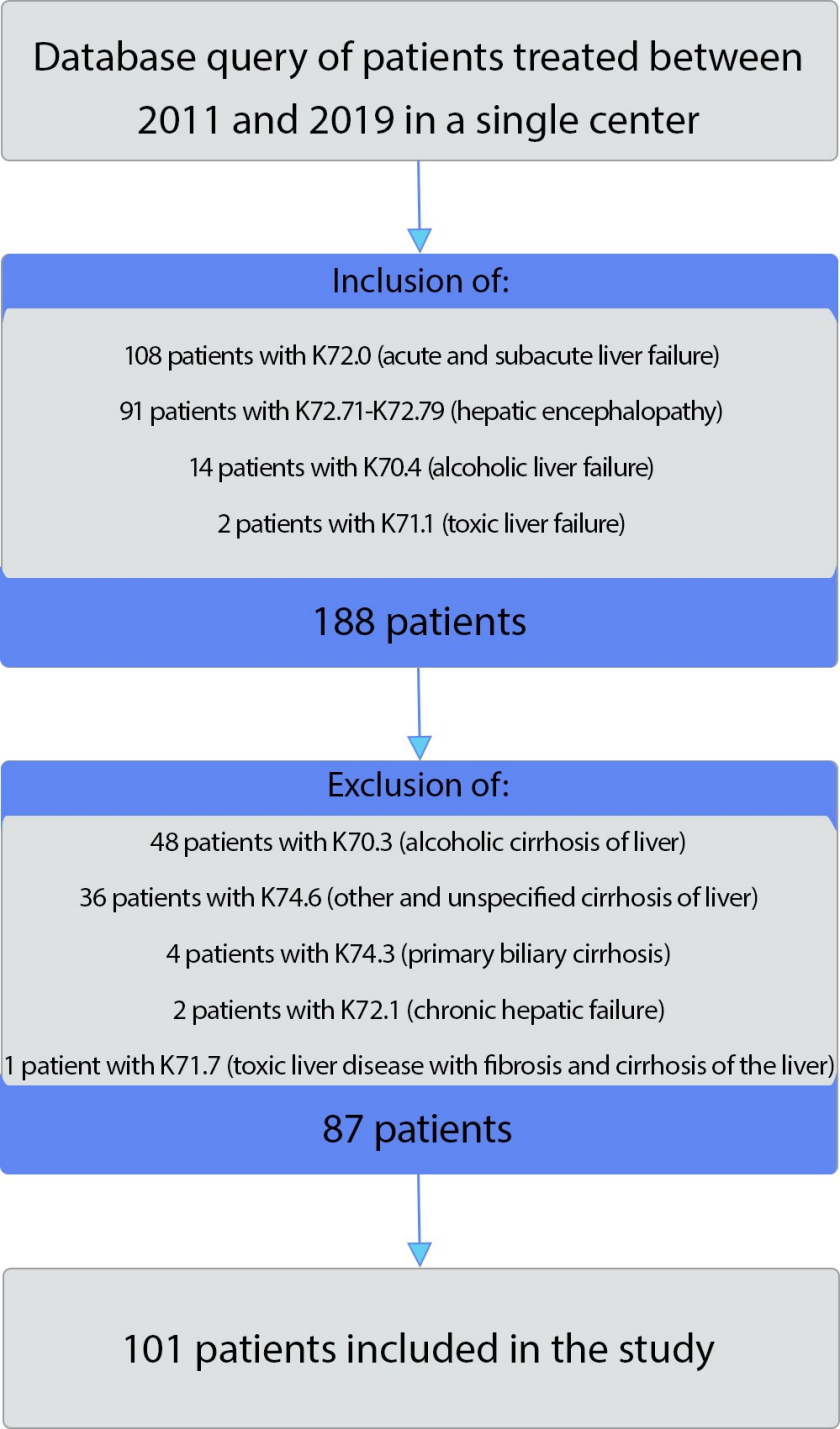
Inclusion and exclusion criteria. 188 patients with acute liver failure were identified in a database query of patients in whose treatment our department at the *RWTH Aachen University Hospital* has been directly involved between 2011 and 2019. The flowchart illustrates the inclusion and exclusion criteria for further statistical analysis.

In a database query of patients in whose treatment the *Department of Anesthesiology* at the *RWTH Aachen University Hospital* has been directly involved between 2011 and 2019, 188 patients with ALF were identified based on ICD 10 codes and included anonymously for retrospective analysis. The evaluation of patient data was approved by the ethics committee of *RWTH Aachen University* (EK291/13) and was carried out in accordance with the Health Data Protection Act NW (§ 6 para. 2 *GDSG NW [17]*). Since it is a strictly retrospective evaluation and data was anonymized prior to analysis by removing any identifiers of individuals to minimize the risk of re-identification individual-level informed consent was not necessary and not required by the ethics committee. No additional tests for research purposes were performed. The inclusion and exclusion criteria are shown in Fig 1. A total of 188 cases were examined, which suffered from ALF or hepatic encephalopathy (ICD-10 K72.0, K70.4, K71.1, K72.71-K72.79) during the respective stay. All patients with pre-existing cirrhosis or chronic liver failure (n = 87) were excluded. Of the remaining 101 patients, one patient had to be excluded after outlier analysis (see statistics), so 100 patients were eventually included in the analysis of this study. A possible association between ALF and secondary acute myocardial injury was investigated; therefore, it was tested whether patients with ALF showed increased markers for myocardial injury (CK, CKMB) and their impact on mortality, taking into account possible confounders in a multivariate regression analysis as described below. Additionally, the influence of pre-existing medical conditions and in-hospital complications was analyzed. The severity of ALF was assessed using the *Model for End-Stage Liver Disease* (MELD) score. In a meta-analysis of 23 studies with a total of 2152 patients, McPhail *et al*. showed that the *Kings College Criteria* (KCC) and the MELD score are comparable in predicting mortality in ALF, but the MELD score has an advantage in non-acetaminophen-associated ALF (NAALF). As our study population includes only two patients with toxic liver failure, we chose the MELD score over the KCC to quantify the severity of ALF [18–21]. For the evaluation the respective maximum values were chosen.

### 2.7 Statistics

Statistical analysis and presentation of data were performed using *GraphPad Prism 8* (version 8.0.2, *GraphPad Software*, *Inc*., La Jolla, CA), *SAS 9*.*4* (*SAS Institute*, Cary, NC) and *JMP 14*.*1* (*SAS Institute*, Cary, NC).

As a null hypothesis, it was assumed that animals from the BDL group are equally as prone to myocardial injury as sham-operated animals that served as controls. Normality and homoscedasticity of data were confirmed using diagnostic plots (residuals and quantile plots of logarithmized data). A one-sided *Dunnett test* was used to analyze whether mediator levels in the treatment groups were higher than those in controls. The null hypothesis was rejected when p<0.05.

For the dataset gained from the retrospective analysis of patient data, the *Mahalanobis distance method* was used for multivariate outlier analysis [22]. One patient was excluded accordingly.

To identify group differences, a *Pearson chi*^*2*^ *test* was performed for nominal variables, and a two-sided t-test was performed for metric variables with a given normal distribution. In the case of heteroscedasticity, the t-test was performed with *Welch’s correction*. For markers of myocardial injury, a one-sided t-test was performed, also with *Welch’s correction* in cases of heteroscedasticity. If the data were not normally distributed, a nonparametric test was chosen (e.g., *Mann-Whitney*).

To identify potential risk factors for in-hospital mortality, multivariate nominal logistic regression was performed. The variables remaining in the model were determined using p-value-based backward elimination. The threshold value for remaining in the model was p = 0.1. Prior to regression analysis, variables were tested for collinearity and excluded accordingly. As independent numeric risk factors, age, hours of artificial respiration, MELD score, CK, CKMB, serum Na+, white blood cell count (WBC), C-reactive protein (CRP), procalcitonin (PCT) and prothrombin time (PTT) were tested. In addition, the following factors were analyzed in the same way: the nominal variables *stroke*, *acute myocardial injury*, *cardiac arrest*, *heart failure*, *therapeutic catheter intervention*, *major adverse cardiac events* (MACE; consisting of the aforementioned variables), *atrial fibrillation or flutter*, *supraventricular tachycardia*, *cerebral bleeding* and *sepsis*, as well as the influence of pre-existing medical conditions such as *diabetes*, *ischemic heart disease*, *heart failure*, *cerebrovascular disease* and *kidney disease*. The aforementioned variables were extracted from ICD-10 codes, and a list of the codes used can be found in the (S3 Table). The effect of the individual variables was assessed using the *likelihood ratio test*. p<0.05 was considered as significant.

To verify the predictive value of the identified variables and to set their thresholds, a *receiver operating characteristic* (ROC) analysis was performed and plotted as specificity and sensitivity versus criterion value, as it allows us to see the sensitivity and specificity for any cutoff value [23]. As thresholds for specificity and sensitivity, a respective value > 90% was used. The positive and negative predictive values for the study population on hand were calculated by means of a standard contingency table [24].

## 3 Results

### 3.1 Analysis of clinical data

The retrospective analysis included 100 patients with ALF with an average age of 53.1 ±17 years (43 males, 57 females) hospitalized between 2011 and 2019 in a single center. Table 1 shows the patient demographics and average laboratory values. 52 of 100 patients (52%) died during their stay in the hospital and showed a significantly higher mean MELD score (surviving: MELD = 29.6 vs. deceased: MELD = 34, p = 0.002). The deceased were 7.4 years older on average (p = 0.03); 22% were men, and 30% were women. Of 100 patients, 87 had acute and subacute hepatic failure with no specified etiology or hepatic encephalopathy without chronic liver disease, 11 had alcoholic liver failure, and 2 suffered from toxic liver failure. The respective etiology had no impact on mortality.

**Table 1:** Baseline demographics and laboratory values.

	**male**		**female**		**total**			
	**n**	**share**	**n**	**share**	**n**	**share**	**group difference (p-values)**	**impact on mortality (p-values)**
total	43	43.0%	57	57.0%	100	100.0%	0.884	0.884
survived	21	21.0%	27	27.0%	48	48.0%		
deceased	22	22.0%	30	30.0%	52	52.0%		
	**survived**		**deceased**		**total**			
	**mean**	**SD**	**mean**	**SD**	**mean**	**SD**	**group difference (p-values)**	**impact on mortality (p-values)**
age [years]	49.3	16.6	56.7	16.8	53.1	17.0	0.029	> 0.1
length of stay [days]	30.8	30.4	22.5	27.5	26.5	29.1	0.014	excl.
hours of artificial respiration [hours]	167.6	371.4	208.8	350.3	189.0	359.3	0.0008	> 0.1
MELD (model for endstage liver disease)	29.6	8.1	34.0	7.2	31.9	7.9	0.002	0.051
INR (international normalized ratio)	2.6	1.2	4.3	3.5	3.5	2.8	0.002	
creatinine [mg/dl]	3.2	3.2	2.7	1.3	2.9	2.4	0.363	
total bilirubin [mg/dl]	13.0	16.7	12.2	10.6	12.6	13.8	0.697	
ALT [U/l] (alanine aminotransferase)	1172.7	1654.2	1193.5	1588.5	1183.5	1612.2	0.165	> 0.1
AST [U/l] (aspartate aminotransferase)	1764.7	2568.7	2240.9	3021.9	2012.3	2809.6	0.400	excl.
CKMB [μg/l] (creatine kinase-MB isoenzyme)	51.7	65.6	176.2	336.5	119.6	258.7	0.023	0.013
CK [U/l] (creatine kinase)	555.9	1162.6	824.2	1853.4	694.10	1555.4	0.197	> 0.1
CRP [mg/l] (C-reactive protein)	119.5	96.9	139.2	97.6	129.6	97.2	0.290	> 0.1
PCT [μg/l| (procalcitonin)	13.5	23.5	11.2	17.1	12.3	20.2	0.249	> 0.1
WBC [G/l] (white blood cell count)	17.6	14.3	26.3	14.9	22.1	15.2	0.0005	0.003
PTT [sec| (pro-thrombin time)	66.1	30.6	79.7	30.7	73.1	31.3	0.016	> 0.1
Na+ [mmol/l] (serum sodium)	133.8	36.8	137.8	31.6	135.9	34.1	0.335	> 0.1

**Table 1.** Shown are the baseline demographics and laboratory values with individual numbers and percentage of the total for nominal values and mean ± standard deviation (SD) for metric values, as well as the respective p-values. WBC (p = 0.003) and CKMB (p = 0.013) showed a significant impact on mortality. The impact on mortality represents the results of the multivariate logistic regression analysis. p<0.05 was considered significant. AST and length of stay had to be excluded (excl.) due to collinearity. The necessary condition for remaining in the model was p<0.1.

**Fig 2:**
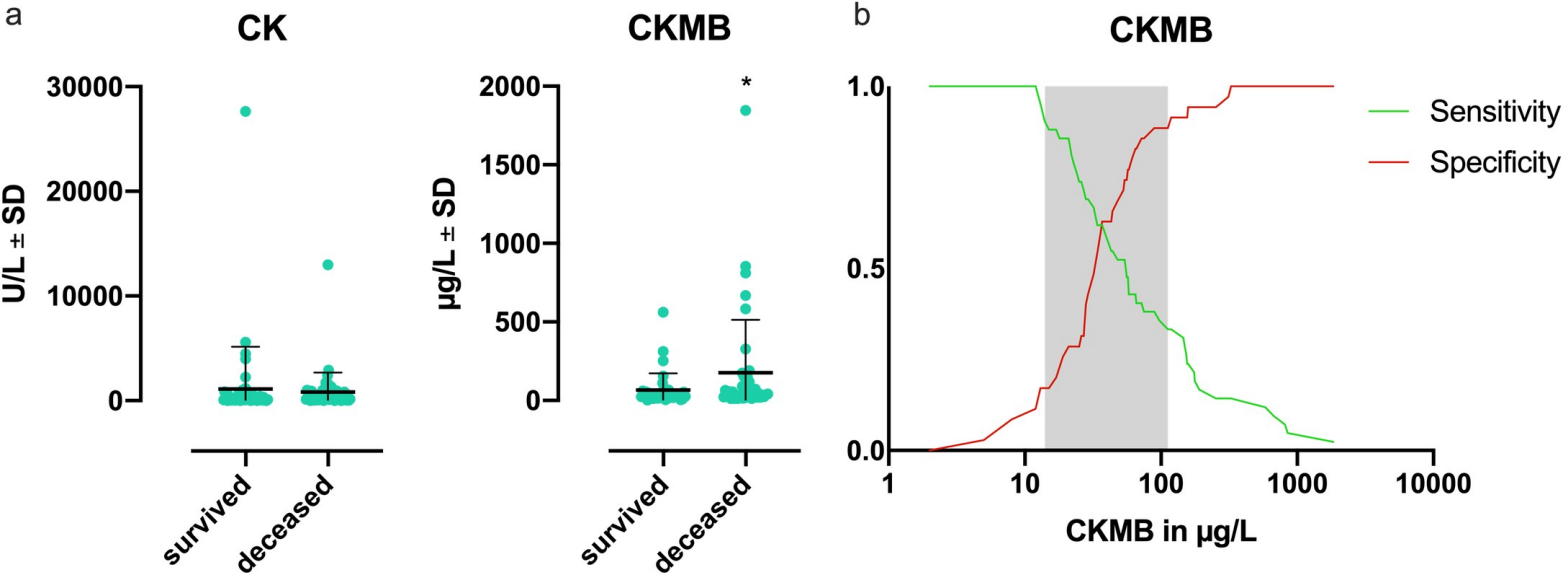
Patients who died of acute liver failure showed significantly higher CKMB values than those who survived. CKMB also has predictive value concerning mortality in ALF. **(a)** Creatine kinase (CK) and creatinine kinase-MB isoenzyme (CKMB) in relation to mortality. Patients who died in the course of acute liver failure (ALF) exhibited significantly higher CKMB values than survivors (*p = 0.023, surviving n = 36, deceased n = 42). CK values showed no difference (p = 0.2, surviving n = 49, deceased n = 51). Shown are the means ± SDs, p<0.05 was considered significant. **(b)** Plotted is the result of the receiver operating characteristic (ROC) analysis (AUC = 0.64, p = 0.006) as specificity and sensitivity versus CKMB values. CKMB>112 μg/L has a positive predictive value (PPV) of 66.7% and a negative predictive value (NPV) of 91.4%.

Diabetes mellitus was the only pre-existing medical condition that had an effect on mortality (p = 0.009). The prevalence of ischemic heart disease or heart failure had no impact (Table 2). However, we observed an increase of 49% in cardio-specific diagnoses during the stay within the entire collective (26% on admission, 75% on discharge, Δ = 49%). In line with that, the analysis of severe complications during the stay identified MACEs as a major contributing factor concerning mortality (p = 0.037, Table 3), as well as sepsis (p<0.002 Table 3). Moreover, the deceased patients exhibited significantly increased levels of CKMB in comparison to those who survived (Fig 2A; p = 0.023). The multivariate regression analysis identified CKMB and WBC as independent predictors of mortality in ALF (CKMB: p = 0.013; WBC: p<0.003). A subsequent ROC analysis (AUC = 0.64, p = 0.006; S1 Fig) revealed a positive predictive value of 66.7% for mortality and a negative predictive value of 91.4% for CKMB values > 112 μg/L (Fig 2B).

**Table 2:** Medical history.

	survived		deceased		total			
pre-existing medical condition	n	share	n	share	n	share	group difference (p-values)	impact on mortality (p-values)
diabetes, all forms	0	0.0%	5	5.0%	5	5.0%	0.028	0.009
ischemic heart disease	1	1.0%	1	1.0%	2	2.0%	0.954	> 0.1
heart failure	4	4.0%	2	2.0%	6	6.0%	0.345	> 0.1
cerebrovascular disease	0	0.0%	1	1.0%	1	1.0%	0.334	> 0.1
kidney disease	8	8.0%	6	6.0%	14	14.0%	0.460	> 0.1

Table 2 shows the pre-existing medical conditions with cardiovascular relevance with individual numbers, their percentages and the respective p-values. Pre-existing diabetes had a significant impact on mortality (p = 0.009). The impact on mortality represents the results of the multivariate logistic regression analysis. p<0.05 was considered significant, and the necessary condition for remaining in the model was p<0.1.

**Table 3:** In-hospital complications.

	survived		deceased		total			
	n	share	n	share	n	share	group difference (p-values)	impact on mortality (p-values)
major adverse cardiac event	10	10.0%	20	20.0%	30	30.0%	0.055	0.037
stroke	1	1.0%	3	3.0%	4	4.0%	0.347	
acute myocardial ischemia	2	2.0%	3	3.0%	5	5.0%	0.713	
cardiac arrest	2	2.0%	8	8.0%	10	10.0%	0.062	
heart failure	8	8.0%	11	11.0%	19	19.0%	0.568	
therapeutic catheter intervention	1	1.0%	1	1.0%	2	2.0%	0.954	> 0.1
cerebral bleeding	0	0.0%	1	1.0%	1	1.0%	0.334	> 0.1
acute anemia due to bleeding	18	18.0%	34	34.0%	52	52.0%	0.005	0.07
sepsis, all categories	13	13.0%	34	34.0%	47	47.0%	0.0002	0.002
septic shock	10	10.0%	24	24.0%	34	34.0%	0.008	
atrial fibrillation or flutter	4	4.0%	6	6.0%	10	10.0%	0.594	> 0.1
supraventricular tachycardia	2	2.0%	1	1.0%	3	3.0%	0.511	> 0.1
thrombosis or embolism	0	0.0%	4	4.0%	4	4.0%	0.049	0.049

**Table 3.** In-hospital complications are displayed with individual numbers, their percentages and the respective p-values. MACE (p = 0.037), sepsis (p = 0.002) and thrombosis or embolism (p = 0.049) had a significant impact on mortality. The impact on mortality represents the results of the multivariate logistic regression analysis. p<0.05 was considered significant, and the necessary condition for remaining in the model was p<0.1.

### 3.2 Animal experiments

The rats showed both a significant increase in markers of liver damage (S2 Fig) and clear histologically visible damage in liver tissue (S3 Fig).

#### 3.2.1 Rats that died early after BDL show increased serum markers of myocardial injury and histologic changes in the myocardium

**Fig 3:**
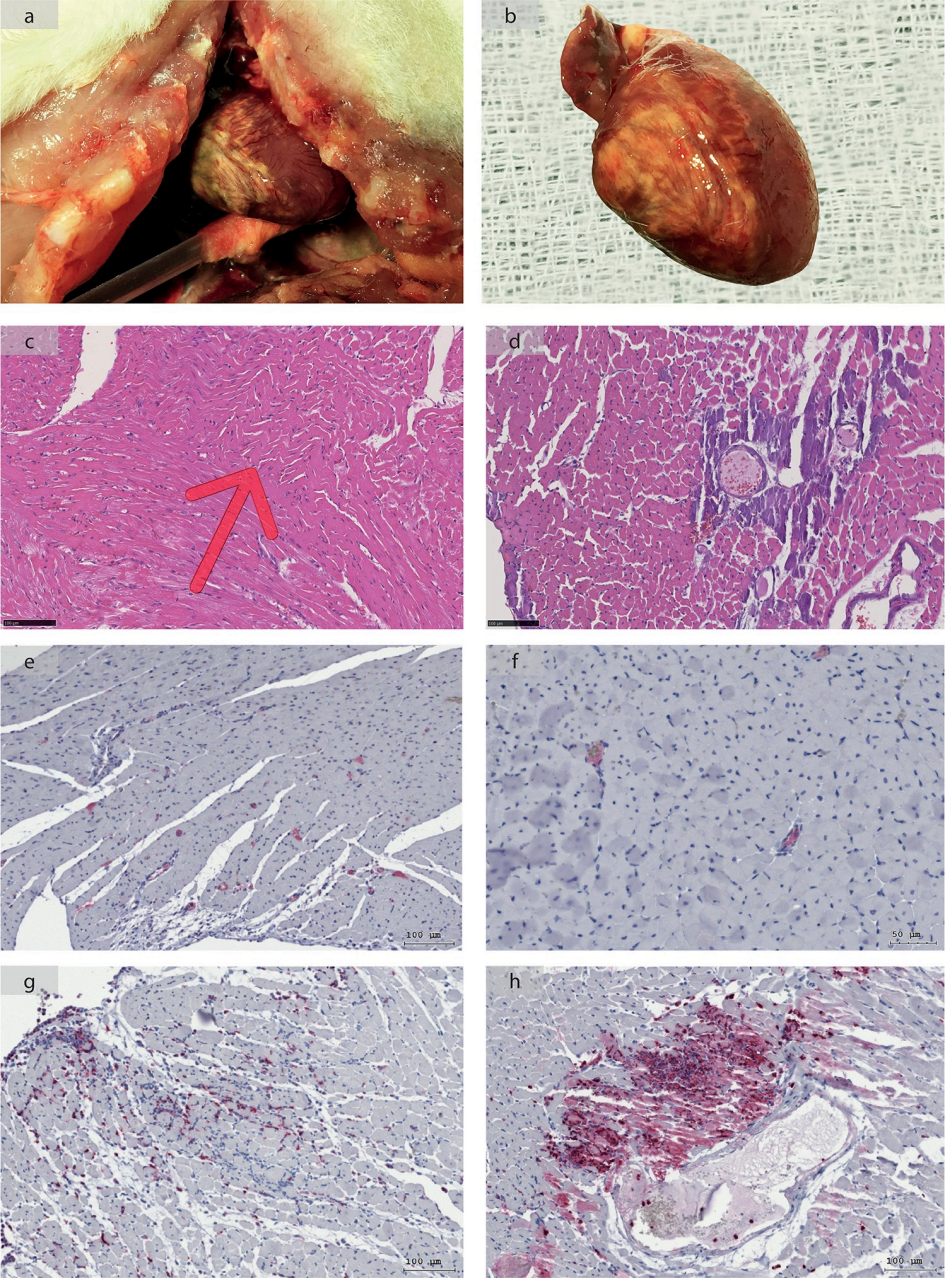
Macroscopic and histologic examination of heart and heart tissue of animals that died early after Bile Duct Ligation (BDL) showed signs of myocardial injury. **(a, b)** The hearts of early deceased animals showed yellow demarcated areas. **(c, d)** *Hematoxylin and eosin staining* (HE) of these animals showed “wavy fibers” (red arrow) in addition to perivascular lesions. The myocardium appeared loosened in general. **(e, f)** CD41 staining showed microthrombi (red) in the capillary system in the myocardium of rats that died early after BDL. **(g)** Myeloperoxidase (MPO) staining showed diffuse spreading of neutrophil granulocytes (dark red) in myocardial tissue as well as **(h)** neutrophil granulocytes infiltrating perivascular lesions in rats that died early after BDL.

In the course of the original experiments, an above-average dropout rate of 20% (8/40) was observed. The presumed cause of death of the animals, determined by observation and autopsy, showed the following distribution: 5 animals died due to myocardial infarction, 2 were euthanized due to a reduced general condition, and 1 died in the course of operative complications. In the five animals that supposedly died from MI, macroscopically visible myocardial lesions were seen during the autopsy (Fig 3A and 3B), and 1 animal with MI had foamy secretion in the lungs.

Histological examination of these animals showed perivascular lesions in the HE-stained sections, such as wavy fibers (Fig 3C and 3D). Immunohistochemistry revealed microthrombi (CD41, Fig 3E and 3F) in the capillary area of the myocardium. No larger fibrotic areas were seen in EVG stains.

**Fig 4:**
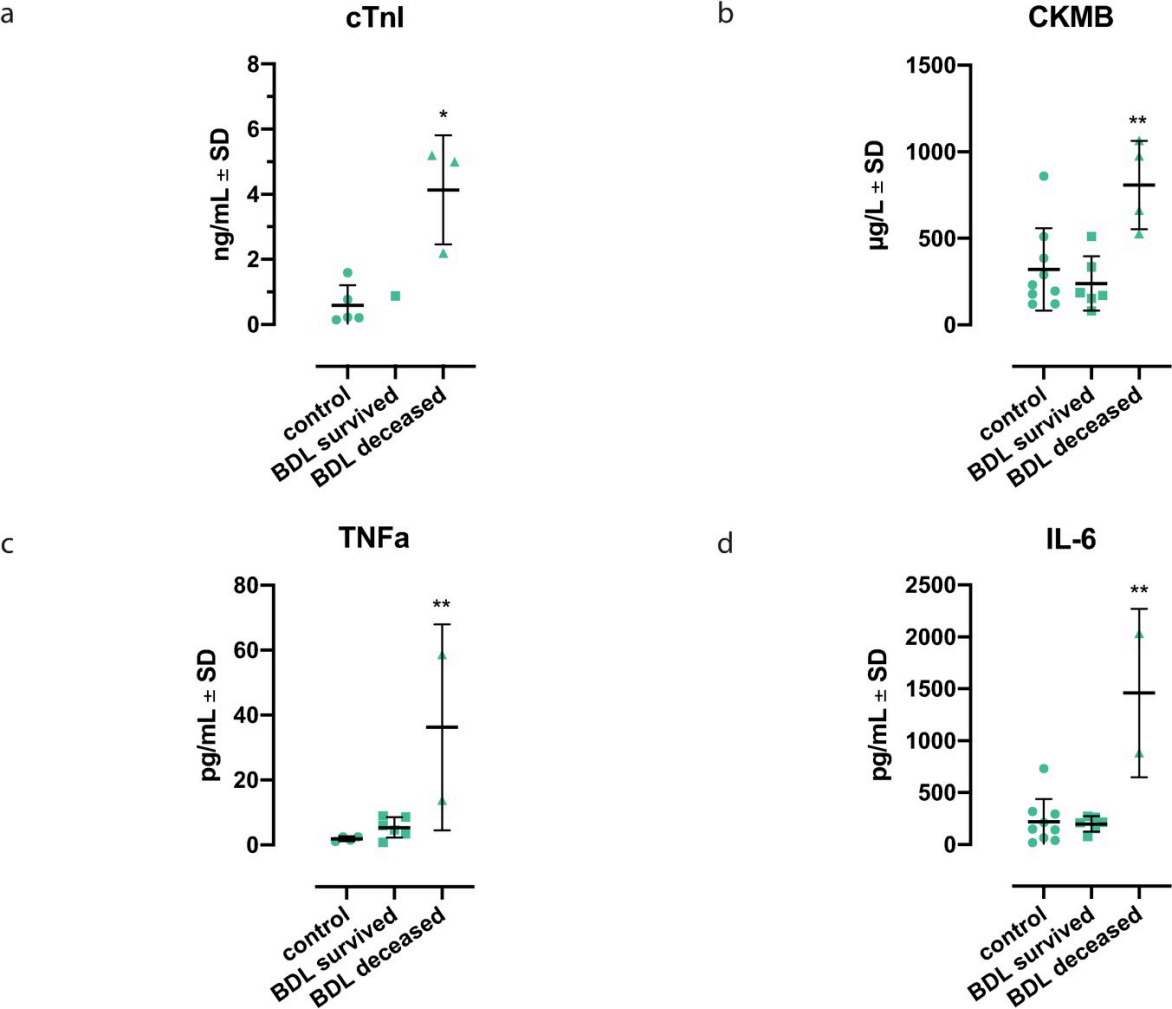
Rats that died early after Bile Duct Ligation (BDL) exhibited higher levels of cardiac troponin I (cTnI), creatine kinase-MB isoenzyme (CKMB), tumor necrosis factor α (TNFα) and interleukin 6 (IL-6) than sham-operated controls 31 days after BDL. Significantly higher **(a)** cTnI values (*p = 0.011, control n = 5, BDL survived n = 1, BDL deceased n = 3), **(b)** CKMB values (**p = 0.008, control n = 9, BDL survived n = 6, BDL deceased n = 4), **(c)** TNFα values (**p = 0.003, control n = 4, BDL survived n = 6, BDL deceased n = 2) and **(d)** IL-6 (**p = 0.006, control n = 9, BDL survived n = 6, BDL deceased n = 2) were observed in rats that died early after BDL compared to respective controls. Displayed are the mean values ± SDs, and p<0.05 was considered significant.

Additional biochemical analysis of the blood samples demonstrated significantly higher cTnI and CKMB values in rats that died early after BDL compared to corresponding controls (Fig 4A and 4b; cTnI: p = 0.011, CKMB: p = 0.008).

#### 3.2.2 Deceased bile duct ligated rats develop an early acute inflammatory state

TNFα and IL-6 levels in rats that died early after BDL were significantly increased compared to controls (Fig 4C and 4D, TNFα: p = 0.003, IL-6: p = 0.006). Moreover, diffuse aggregates of neutrophilic granulocytes in the myocardial tissue (MPO, Fig 3G and 3H) and neutrophilic infiltration of the perivascular lesions already seen in HE staining were observed.

#### 3.2.3 One rat that died early after BDL showed a reduction in radial strain and ECG abnormalities

**Fig 5:**
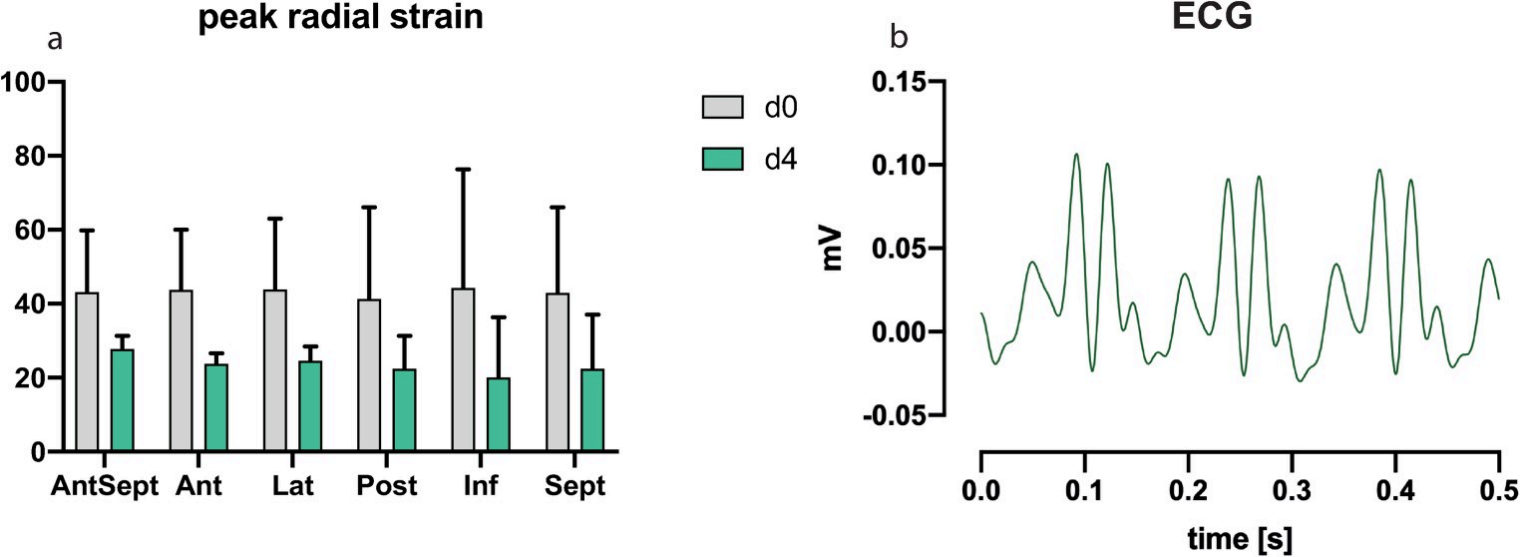
(a) Peak radial strain in a rat that died early after BDL was reduced in comparison to baseline prior to intervention, and (b) ECG abnormalities that were consistent with an intraventicular block were observed in the same animal.

TTE before euthanasia was only possible in 2 animals. One animal showed reduced radial strain at day 4 and cardiac conduction disorder in the form of an intraventricular block in the ECG shortly after BDL (Fig 5).

No significant differences in basic echocardiographic measurements and hemodynamics could be found during the first surgery between the animals that survived and those that died related to myocardial injury.

## 4 Discussion

We investigated the hypothesis that secondary acute myocardial injury occurs in the context of ALF. Therefore, we retrospectively analyzed 100 patients suffering from ALF and 8 animals that died early after BDL and controls. This hypothesis was supported by a high incidence of MACEs and increased levels of CKMB, as well as CKMB as an independent risk factor for mortality in patients with ALF. Furthermore, the animals that died early after BDL demonstrated several signs of diffuse myocardial injury.

Common complications in ALF are cerebral edema, coagulopathy with associated bleeding and sepsis and multiorgan failure [5]. While there are repeated publications reporting cardiac complications and causes of death in ALF, this observation has yet been given little consideration [7, 8, 12, 25]. Hence, it also seems likely that the incidence of cardiac causes of death is underestimated since it might be overlooked.

Our present analysis has now shown not only that there has been a 49% increase in cardio-specific diagnoses in patients with ALF during their stay in the hospital but also that the occurrence of MACEs is a critical factor for mortality that is almost as important as sepsis (MACE: p = 0.037 vs. sepsis p = 0.002 Table 3).

To exclude that the observed increased rate of MACEs in the deceased patients was due to a higher risk profile, the pre-existing medical conditions, in particular cardiovascular risk factors, of both groups (surviving and deceased) were analyzed. It was found that only diabetes (p = 0.009) but not ischemic heart disease or heart failure had an impact on mortality (Table 2). Thus, it seems likely that myocardial injury occurs within the course of ALF. This is further supported by the fact that the deceased patients had a more severe ALF (MELD score: 34 vs. 29.6, p <0.002, Table 1) and, on the other hand, significantly higher CKMB levels than those who survived (Fig 2A). As CKMB has high specificity as a marker for cardiac tissue injury [26], it is reasonable to conclude that structural damage to the myocardium is associated with ALF. Since CKMB returns to baseline within 36 to 48 hours [27], it is also possible that the available CKMB measurements do not reflect all myocardial lesions and therefore underestimate the actual incidence. Nonetheless, CKMB was identified as an independent predictor of mortality in ALF (p = 0.013), showing a positive predictive value of 66.7% and a negative predictive value of 91.4% for CKMB values above 112 μg/L (Fig 2B). The fact that the CK and CKMB values were not measured at a standardized time point after the onset of ALF must be considered a limiting factor, at least with regard to the time course of secondary myocardial injury. However, we would argue that the respective maxima allow a better answer to the question whether secondary myocardial injury occurs within ALF at all.

This hypothesis is further supported by our observations from animal studies. The fact that rats after BDL show liver injury both histologically and in biochemical analysis as well as significantly increased inflammatory markers confirmed that ALF was successfully induced. As mentioned earlier, above-average mortality in the first few days after BDL was found to be associated with macroscopically visible myocardial injury. Georgiev *et al*. have already reported high mortality rates after BDL (9/63, 14.3%) [28] but have not yet investigated this issue from a cardiovascular perspective. From their former studies of the BDL model in mice, the ALF is known to peak on days 2–3, which is also consistent with the observed complications after BDL in our model [28]. Infectious complications may occur after BDL but usually considerably later [29, 30]. At this early stage after BDL, we do not assume that the animals died of fulminant sepsis, although this cannot be excluded with certainty. However, we did see macroscopically visible myocardial lesions sustained by significantly increased CKMB and cTnI levels.

In contrast to Audimoolam *et al*. [8], the significantly increased cTnI and CKMB values and histologically visible myocardial lesions in rats that died early after BDL (Figs 3 and 4A and 4B) indicate that not only myocardial metabolic stress but also actual structural damage occurs in the course of ALF in rats. This was also demonstrated by HE staining of myocardial tissue (Fig 3C and 3D). The observed wavy fibers are consistent with myocardial infarction [31], whereas the perivascular lesions are rather atypical, supporting the idea of a mechanism of injury originating from the vascular system. Furthermore, one animal showed foamy secretion in the lungs during autopsy, most likely pulmonary edema, which is consistent with congestive heart failure. In at least one animal, a reduction in left ventricular function in the strain analysis in combination with an interventricular conduction block was observed. This can be interpreted as an expression of functional myocardial impairment that may lead to pulmonary congestion.

It must of course be admitted that the number of animals in our experiments was comparatively small and therefore rather indicative. Nevertheless, this provides both support for the phenomenon described in the patient data and an outlook on possible pathomechanisms.

Moreover, the analysis of in-hospital complications in human patients showed an incidence of 24% for septic shock within the deceased patients (34% overall; Table 3). By definition, septic shock includes not only cellular and metabolic abnormalities but also impairments of the cardiovascular system [32]. Based on our observations, it is conceivable that the definition of septic shock may obscure possible cardiac causes of death secondary to ALF rather than sepsis. In 2019, Seymour *et al*. introduced four new clinical phenotypes of sepsis (alpha, beta, gamma and delta), whereby the delta type is described as a combination of liver failure and septic shock. This delta type has by far the highest mortality, at 40% [33]. The combination of a heart predamaged by ALF in a hyperdynamic circulatory condition and increased oxygen consumption in the course of sepsis could be the key to the reported high mortality in delta-type sepsis [34].

Although the *Sepsis-3 Definition [32]* the *Systemic Inflammatory Response Syndrome* (SIRS) is no longer relevant for the diagnosis of sepsis due to its lack of sensitivity and specificity, it is still an integral component of the pathophysiology of ALF where high levels of circulating proinflammatory cytokines are observed [35, 36]. This is compatible with the increased levels of TNFα and IL-6 measured in our study (Fig 4C and 4D) and may lead to endothelial dysfunction. One possible mechanism proposed stated that proinflammatory cytokines such as TNFα or IL-6 reduce the phosphorylation of eNOS by increasing oxidative stress (ROS) and thus also have an inhibitory effect on nitric oxide (NO) production [37, 38]. Huet *et al*. also showed that plasma from patients suffering from septic shock *in vitro* causes an increased production of ROS [39]. This leads to the conclusion that SIRS in the context of ALF could trigger endothelial dysfunction of the heart in a humoral way. The reduction of NO-dependent effects, such as vasodilatation, modulation of platelet aggregation and adhesion of leukocytes and monocytes, results in increased vasoconstriction with possible ischemia, thrombosis and increased inflammatory processes in the endothelium [40, 41]. In accordance with this, CD41 staining actually showed thrombi in the microcirculation of rats that died early after BDL (Fig 3E and 3F). The perivascular lesions seen in Figs 3D and 2H may be due to cytotoxic effects in the context of endothelial dysfunction (due to ROS, peroxynitrite and monocytes).

However, adverse cardiac effects in the context of a systemic inflammatory response are probably not exclusively limited to ALF. Flurian *et al*. showed an inverse correlation of *endothelin-1* (ET-1) as a marker of endothelial dysfunction with left and right ventricular function, indicating that endothelial dysfunction might also contribute to myocardial injury in sepsis alone [42].

Furthermore, activation of the *myosin light chain kinase* (MLCK) pathway by cytokines such as TNFα leads to endothelial barrier dysfunction [43, 44]. Additionally, TNFα induces apoptosis in endothelial cells by recruiting *caspase-8* via activation of *TNF receptor 1* (TNFR1) [45–47]. Therefore, extravasation of proinflammatory cytokines and leukocytes (e.g., neutrophils and macrophages) occurs. In tissue, TNFα likewise affects cardiomyocyte apoptosis [48]. It was also demonstrated that apoptotic endothelial cells have both a pro-adhesive effect on platelets and neutrophils as well as pro-coagulative properties [49–51]. Therefore, the direct proinflammatory and proapoptotic signaling effects of TNFα may also have led to the observed perivascular lesions and microthrombi (Fig 3D and 3F).

Apart from extravasation due to endothelial barrier dysfunction, activation of the endothelium by TNFα and other cytokines results in luminal expression of *intercellular adhesion molecules* (ICAMs) and selectins and thus in adhesion and transmigration of neutrophils [52]. This is consistent with the fact that we observed diffuse dissemination of neutrophils in tissue in the histological examination, such as increased accumulation in perivascular lesions (Fig 3G and 3H). Activated neutrophils degranulate and release larger quantities of ROS, causing inflammatory tissue injury, which in turn leads to further chemotaxis of neutrophils and monocytes [53].

Since ligature of the CBD leads to a considerable early increase in circulating bile acids, it is also conceivable that direct bile acid signaling has an effect on the myocardium. It is already known that bile acids have a negative chronotropic effect on the heart and are also potentially cardiotoxic [54, 55]. Further studies have identified two receptors that play a prominent role in the signal transduction cascade of bile acids, the *farnesoid-X receptor* (FXR) and *Takeda G protein-coupled receptor 5* (TGR5) [56, 57]. Both FXR and TGR5 are expressed in the vascular system (FXR) and in cardiomyocytes (FXR and TGR5). Furthermore, it was demonstrated that FXR has proapoptotic effects via the opening of the *mitochondrial permeability transition pore* (MPTP) and plays a role in myocardial ischemia-reperfusion injury [58]. After activation by bile acids, TGR5 mediates reduced expression of *glycogen synthase kinase-3ß* (GSK3**β**) and increased expression of *protein kinase B* (PKB), both of which are associated with hypertrophy of the heart [59]. In addition, cardiomyocytes derived from rats following BDL show increased activation of proapoptotic signaling pathways with increased cleavage of *poly ADP-ribose polymerase* (PARP) 1 and apoptosis mediating receptor *FasR* [14, 60].

There is also evidence, that cholestasis effects overproduction of NO and related apoptosis of cardiomyocytes, probably due to nitrosative stress similar to endothelial dysfunction [14, 61].

## 5 Conclusions

The results of clinical data analysis and supportive data derived from a rodent BDL model indicate that MACEs play a pivotal role in mortality in ALF, and the increased CKMB values suggest that this might be due to structural myocardial damage. Furthermore, CKMB was identified as an independent predictor of mortality in ALF. Inflammation, endothelial dysfunction and direct bile acid signaling might be involved in the pathogenesis of secondary myocardial injury in the course of ALF. Further research is required to verify causality and identify the exact pathomechanisms.

## Supporting information

S1 Fig Creatine kinase MB-isoenzyme (CKMB) has predictive value for mortality in Acute Liver Failure (ALF). In the receiver-operating characteristics (ROC) analysis (area under the curve (AUC) = 0.64, p = 0.006) CKMB shows predictive value concerning mortality in ALF.
(TIF)

S2 Fig Acute Liver Failure (ALF) was successfully induced in animals that predeceased early after Bile Duct Ligation (BDL). Rats showed significantly higher levels of (a) aspartate aminotransferase (AST; SH n = 9 vs. BDL n = 4, p = 0.0260), alanine aminotransferase (ALT; SH n = 9 vs. BDL n = 4, p = 0.0475) and (b) total bilirubin (SH n = 4 vs. BDL n = 5, p = 0.0189) 31 days after bile duct ligation (BDL) than respective sham operated controls.
(TIF)

S3 Fig HE stain of liver tissue of rats early deceased after BDL and sham operated animals. Liver tissue of rats that (a+b) deceased early after BDL showed biliary infarcts also referred to as *Charcot-Gombault necrosis* (clusters of necrotic hepatocytes; black arrows), in contrast to livertissue (c+d) of sham-operated controls.
(TIF)

S1 Table ICD 10 and OPS codes. Table containing the ICD 10 and OPS Codes that were used for statistical analysis.
(PDF)

S2 Table Etiology of acute liver failure. Table containing the respective etiology of liver failure and its impact on mortality.
(PDF)

S3 Table Animal data. Table containing animal data.
(XLSX)

S1 File JMP report on laboratory values and baseline demographics. Nominal logistic regression analysis of laboratory values, MELD score, age, length of stay and hours of artificial respiration.
(PDF)

S2 File JMP report on pre-existing medical conditions. Nominal logistic regression analysis of pre-existing medical conditions.
(PDF)

S3 File JMP report on in-hospital complications. Nominal logistic regression analysis of in hospital complications.
(PDF)

S4 File ARRIVE guidelines checklist 2.0 (essential 10). (PDF)
