# Supplementary figures and images for: Acute myocardial injury secondary to severe acute liver failure: A retrospective analysis supported by animal data
Source: PLoS One. 2021 Aug 30;16(8):e0256790. doi: 10.1371/journal.pone.0256790 (PMC8405020; doi:10.1371/journal.pone.0256790)

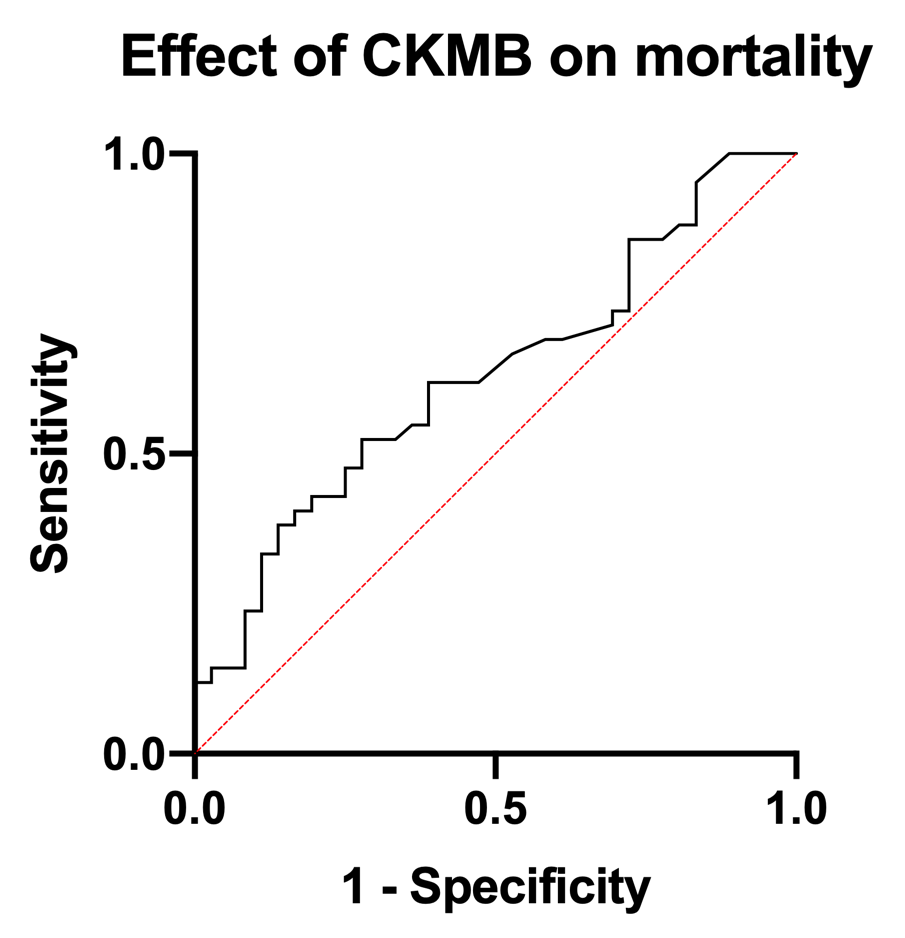

Supplement: S1 Fig — In the receiver-operating characteristics (ROC) analysis (area under the curve (AUC) = 0.64, p = 0.006) CKMB shows predictive value concerning mortality in ALF. (TIF) [file pone.0256790.s001.tif]

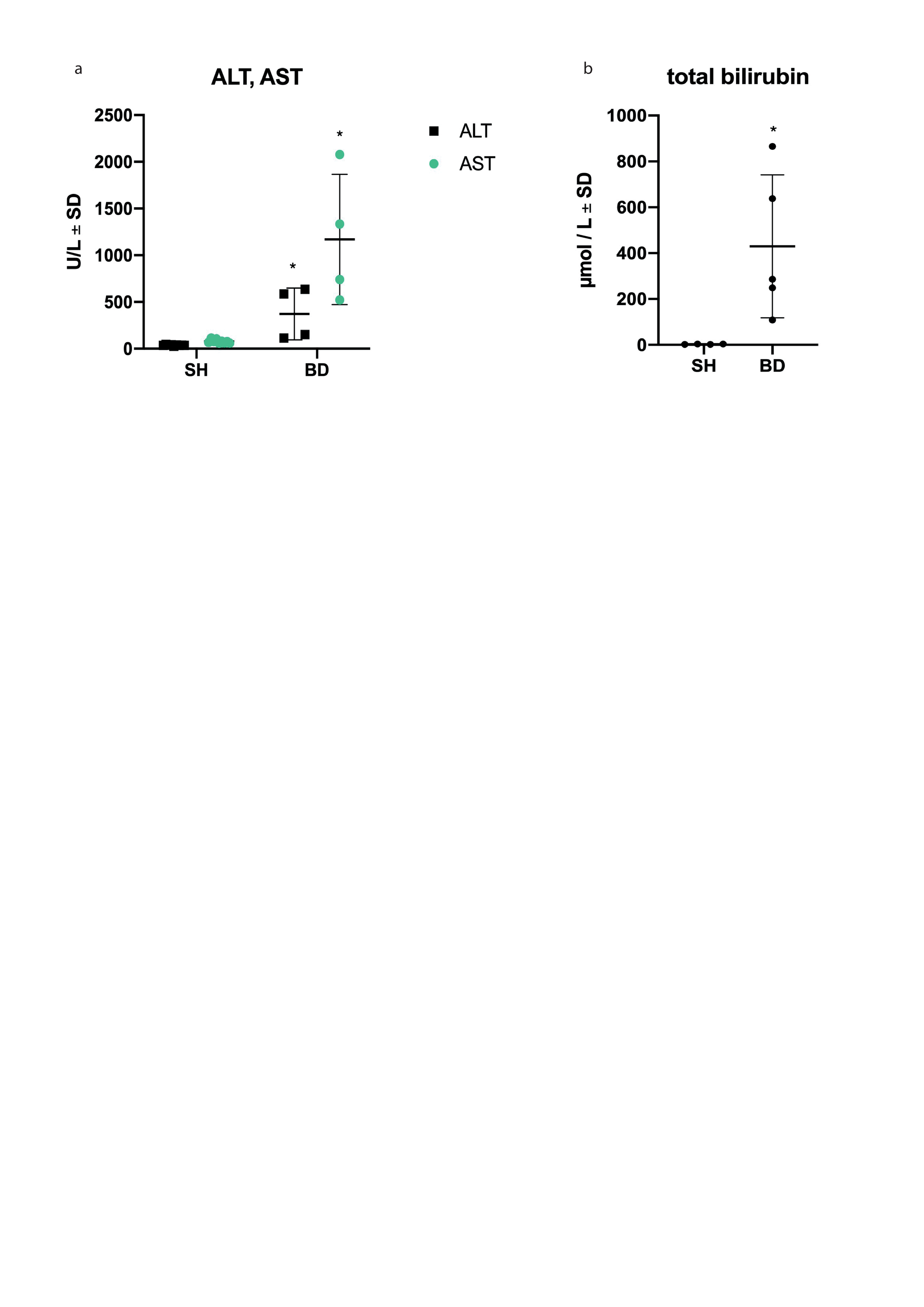

Supplement: S2 Fig — Rats showed significantly higher levels of (a) aspartate aminotransferase (AST; SH n = 9 vs. BDL n = 4, p = 0.0260), alanine aminotransferase (ALT; SH n = 9 vs. BDL n = 4, p = 0.0475) and (b) total bilirubin (SH n = 4 vs. BDL n = 5, p = 0.0189) 31 days after bile duct ligation (BDL) than respective sham operated controls. (TIF) [file pone.0256790.s002.tif]

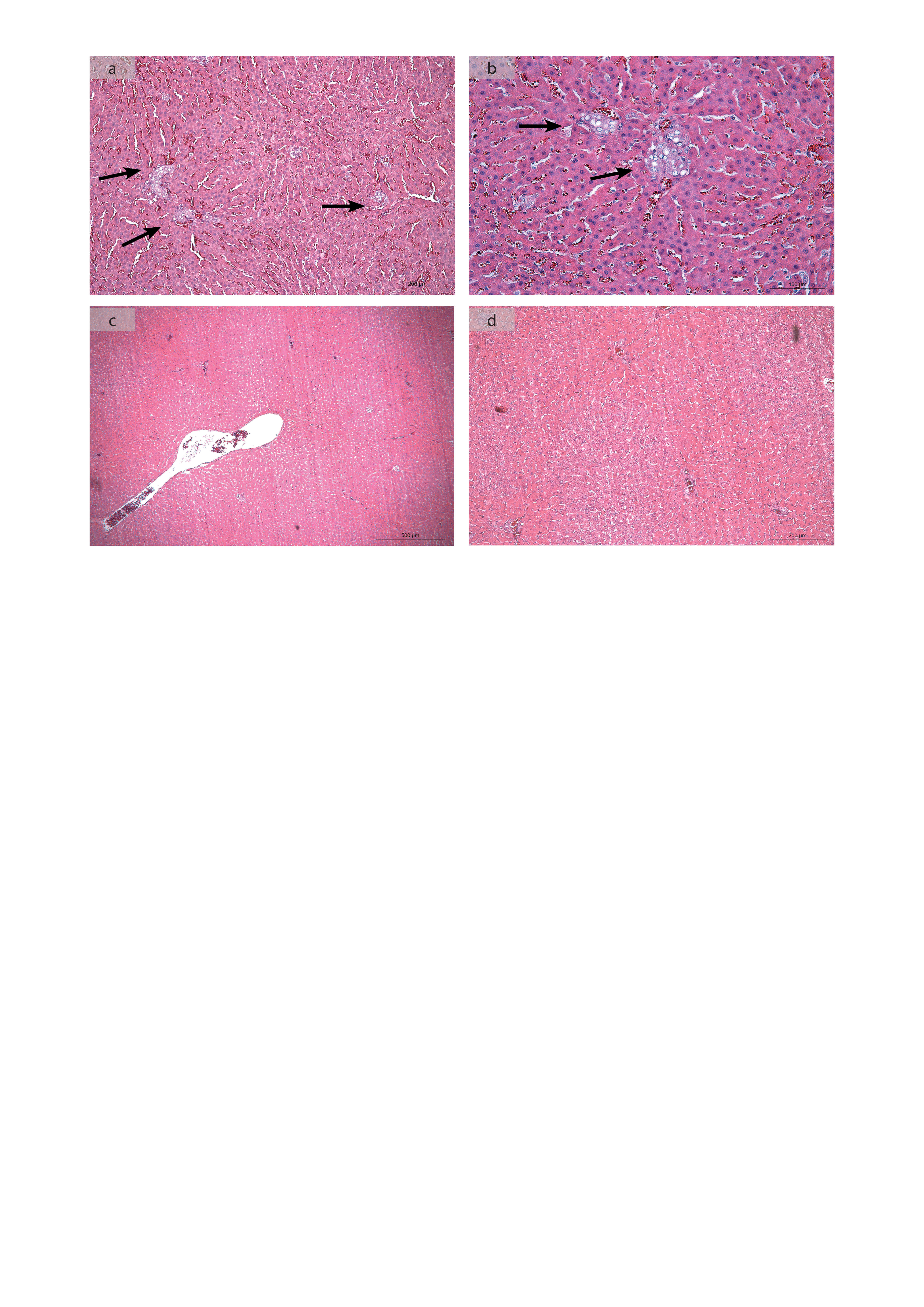

Supplement: S3 Fig — Liver tissue of rats that (a+b) deceased early after BDL showed biliary infarcts also referred to as Charcot-Gombault necrosis (clusters of necrotic hepatocytes; black arrows), in contrast to livertissue (c+d) of sham-operated controls. (TIF) [file pone.0256790.s003.tif]
