# Supplementary material for: Acute myocardial injury secondary to severe acute liver failure: A retrospective analysis supported by animal data
Source: PLoS One. 2021 Aug 30;16(8):e0256790. doi: 10.1371/journal.pone.0256790 (PMC8405020; doi:10.1371/journal.pone.0256790)
Supplement: S1 Table — Table containing the ICD 10 and OPS Codes that were used for statistical analysis. (PDF) [file pone.0256790.s004.pdf]

S1 Table ICD 10 and OPS Codes

Variable	ICD-10/OPS
	**on admission**
pre-existing diabetes, all forms	E10.30, E11.20, E11.40, E11.73, E11.74, E11.90, E13.11, E13.90
pre-existing Ischemic Heart disease	I21.4, I25.0, I25.11, I25.12, I25.13, I25.19
pre-existing heart failure	I50.01, I50.13, I50.14
pre-existing cerebrovascular disease	I61.4, I61.3
pre-existing kidney disease	N17.9, N17.92, N17.91, N17.99, N18.3, N18.4, N18.5, N18.9
	**on discharge**
stroke	I61.4, I61.5, I63.1, I63.3, I63.4, I63.8, I65.2, I65.3, I67.3, I67.7, I67.88, I69.4
acute myocardial ischemia	I20.8, I21.0, I21.4, I23.6
cardiac arrest	I46.0, I46.9
heart failure	I50.01, I50.13, I50.14, I50.9
therapeutic catheter intervention	8-837.00, 8-837.k0, 8-8837.k1, 8-837.m0, 8-837.m1
sepsis, all categories	A40.1, A40.2, A40.8, A41.0, A41.1, A41.4, A41.51, A41.52, A41.58, A41.8, A41.9, T81.4, A49.9, B37.7, R57.2
septic shock	R57.2
cerebral bleeding	I61.4, I61.5, I63.1, I63.3, I63.4, I63.8
acute aneamia due to bleeding	D62
in Hospital SVT	I47.1
in hospital fibrilation or flutter	I48.0, I48.1, I48.3, I48.4, I48.9
in hospital thrombosis or embolism	I26.0, I26.9, I74.2, I74.8
	**on admission or discharge**
acute and subacute liver failure	K72.0
toxic liver failure	K71.1
alcoholic liver failure	K70.4
chronic or cirrhotic liver disease	K70.3, K71.7, K72.1, K74.6, K74.3
hepatic encepahlopathy	K72.71-K72.79
LTX	5-504.0
LTX evaluation with listing	1-920.24, 1-920.34
