# Supplementary material for: Acute myocardial injury secondary to severe acute liver failure: A retrospective analysis supported by animal data
Source: PLoS One. 2021 Aug 30;16(8):e0256790. doi: 10.1371/journal.pone.0256790 (PMC8405020; doi:10.1371/journal.pone.0256790)
Supplement: S2 Table — Table containing the respective etiology of liver failure and its impact on mortality. (PDF) [file pone.0256790.s005.pdf]

S2 Table Etiolgy of acute liver failure

	survived		deceased		total			
etiology	n	share	n	share	n	share	group difference (p-value)	impact on mortality (p-value)
acute and subacute liver failure and hepatic encepahlopathy without chronic liver disease	41	41%	46	46%	87	87%	0.651	> 0.1
toxic liver failure	1	1%	1	1%	2	2%	0.954	> 0.1
alcoholic liver failure	6	6%	5	5%	11	11%	0.645	> 0.1
