# Supplementary material for: Acute myocardial injury secondary to severe acute liver failure: A retrospective analysis supported by animal data
Source: PLoS One. 2021 Aug 30;16(8):e0256790. doi: 10.1371/journal.pone.0256790 (PMC8405020; doi:10.1371/journal.pone.0256790)
Supplement: S1 File — Nominal logistic regression analysis of laboratory values, MELD score, age, length of stay and hours of artificial respiration. (PDF) [file pone.0256790.s007.pdf]

Nominal Logistic Fit for survival

Effect Summary

Source	LogWorth	PValue
WBC (white bloodcell count)	2.535	0.00292
CKMB (MB isoenzyme of creatine kinase)	1.894	0.01277
MELD	1.291	0.05116

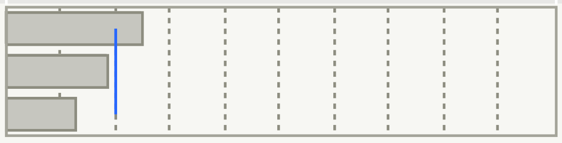

Converged in Gradient, 6 iterations

Iterations

Iter	Objective	Relative Gradient	Norm Gradient
0	53.372332903	4.4407626714	2817.4985569
1	42.240317061	1.6213489339	945.76477603
2	40.663037002	0.6880428113	325.19700596
3	40.393128499	0.1818049351	71.871496007
4	40.375723993	0.0155319622	5.7418001211
5	40.375602769	0.0001171737	0.0431514344
6	40.375602762	6.6812953e-9	2.46139e-6

Whole Model Test

Model	-LogLikelihood	DF	ChiSquare	Prob>ChiSq
Difference	12.678109	3	25.35622	<.0001*
Full	40.375603			
Reduced	53.053711			

RSquare (U)	0.2390
AICc	89.3068
BIC	98.1264
Observations (or Sum Wgts)	77

Fit Details

Measure	Training	Definition
Entropy RSquare	0.2390	1-Loglike(model)/Loglike(0)
Generalized RSquare	0.3751	(1-(L(0)/L(model))^(2/n))/(1-L(0)^(2/n))
Mean -Log p	0.5244	∑ -Log(ρ[j])/n
RASE	0.4180	√ ∑(y[j]-ρ[j])²/n
Mean Abs Dev	0.3519	∑ |y[j]-ρ[j]|/n
Misclassification Rate	0.2208	∑ (ρ[j]≠ρMax)/n
N	77	n

Lack Of Fit

Source	DF	-LogLikelihood	ChiSquare
Lack Of Fit	73	40.375603	80.75121
Saturated	76	0.000000	Prob>ChiSq
Fitted	3	40.375603	0.2499

Parameter Estimates

Term	Estimate	Std Error	ChiSquare	Prob>ChiSq
Intercept	-4.050491	1.3292201	9.29	0.0023*
CKMB (MB isoenzyme of creatine kinase)	0.00526278	0.0030084	3.06	0.0802
WBC (white bloodcell count)	0.06922305	0.0265225	6.81	0.0091*
MELD	0.07227116	0.0383837	3.55	0.0597

For log odds of dead/survived

Covariance of Estimates

Cov

	Intercept	CKMB (MB isoenzyme of creatine kinase)	WBC (white bloodcell count)	MELD
Intercept	1.7668	-0.001	-0.011	-0.045
CKMB (MB isoenzyme of creatine kinase)	-0.001	0.0000	0.0000	-0.000
WBC (white bloodcell count)	-0.011	0.0000	0.0007	-0.000
MELD	-0.045	-0.000	-0.000	0.0015

Effect Likelihood Ratio Tests

Source	Nparm	DF	L-R ChiSquare	Prob>ChiSq
CKMB (MB isoenzyme of creatine kinase)	1	1	6.20064314	0.0128*
WBC (white bloodcell count)	1	1	8.85976901	0.0029*
MELD	1	1	3.80316489	0.0512
