# Supplementary material for: Acute myocardial injury secondary to severe acute liver failure: A retrospective analysis supported by animal data
Source: PLoS One. 2021 Aug 30;16(8):e0256790. doi: 10.1371/journal.pone.0256790 (PMC8405020; doi:10.1371/journal.pone.0256790)
Supplement: S2 File — Nominal logistic regression analysis of pre-existing medical conditions. (PDF) [file pone.0256790.s008.pdf]

Nominal Logistic Fit for survival

Effect Summary

Source	LogWorth		PValue
pre-existing diabetes, all forms	2.036		0.00921

Converged in Gradient, 15 iterations

Iterations

Iter	Objective	Relative Gradient	Norm Gradient
0	69.314718056	2.2384204957	3.6055512755
1	66.478358953	0.822603438	0.8428919454
2	66.056550084	0.466289457	0.2946727037
3	65.919721144	0.2767360068	0.1066706316
4	65.871387218	0.1665683484	0.0390208493
5	65.853858949	0.1007488942	0.014325666
6	65.847444039	0.0610452987	0.0052661822
7	65.845088602	0.0370120501	0.001936789
8	65.84422269	0.0224458681	0.000712433
9	65.84390422	0.0136134213	0.0002620798
10	65.843787072	0.0082568044	0.0000964124
11	65.843743978	0.0050079709	0.000035468
12	65.843728124	0.0030374803	0.0000130479
13	65.843722292	0.0018423232	4.8000565e-6
14	65.843720147	0.0011174251	1.7658417e-6
15	65.843719357	0.0006777525	6.4961678e-7

Whole Model Test

Model	-LogLikelihood	DF	ChiSquare	Prob>ChiSq
Difference	3.390977	1	6.781955	0.0092*
Full	65.843719			
Reduced	69.234697			

RSquare (U)	0.0490
AICc	135.811
BIC	140.898
Observations (or Sum Wgts)	100

Fit Details

Measure	Training	Definition
Entropy RSquare	0.0490	1-Loglike(model)/Loglike(0)
Generalized RSquare	0.0875	(1-(L(0)/L(model))^(2/n))/(1-L(0)^(2/n))
Mean -Log p	0.6584	$\sum -\mathrm{Log}(\rho[j])/n$
RASE	0.4873	$\sqrt{\sum(y[j]-\rho[j])^2/n}$
Mean Abs Dev	0.4749	$\sum \lvert y[j]-\rho[j]\rvert/n$
Misclassification Rate	0.4700	$\sum (\rho[j]\neq\rho\mathrm{Max})/n$
N	100	n

Parameter Estimates

Term		Estimate	Std Error	ChiSquare	Prob>ChiSq
Intercept	Unstable	8.09092061	737.73249	0.00	0.9912
pre-existing diabetes, all forms[not diseased]	Unstable	-8.111974	737.73249	0.00	0.9912

For log odds of dead/survived

Covariance of Estimates

Cov

	Intercept	pre-existing diabetes, all forms[not diseased]
Intercept	544249	-5e+5
pre-existing diabetes, all forms[not diseased]	-5e+5	544249

Effect Likelihood Ratio Tests

Source	Nparm	DF	L-R ChiSquare	Prob>ChiSq
pre-existing diabetes, all forms	1	1	6.7819547	0.0092*
