# Supplementary material for: Acute myocardial injury secondary to severe acute liver failure: A retrospective analysis supported by animal data
Source: PLoS One. 2021 Aug 30;16(8):e0256790. doi: 10.1371/journal.pone.0256790 (PMC8405020; doi:10.1371/journal.pone.0256790)
Supplement: S3 File — Nominal logistic regression analysis of in hospital complications. (PDF) [file pone.0256790.s009.pdf]

# Nominal Logistic Fit for survival

## Effect Summary

| Source | LogWorth | PValue |
|---|---|---|
| sepsis, all categories | 2.804 | 0.00157 |
| major adverse cardiac event | 1.436 | 0.03663 |
| thrombosis or embolism | 1.306 | 0.04945 |
| acute aneamia due to bleeding | 1.146 | 0.07144 |

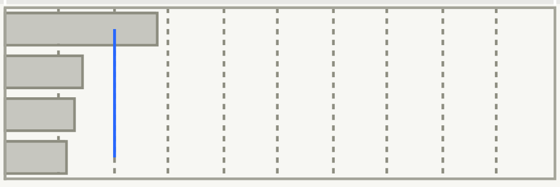

Converged in Gradient, 15 iterations

## Iterations

| Iter | Objective | Relative Gradient | Norm Gradient |
|---|---|---|---|
| 0 | 69.314718056 | 4.8491142631 | 25.079872408 |
| 1 | 56.601301707 | 1.0860404017 | 3.1606947092 |
| 2 | 55.916820808 | 0.4579732885 | 0.3476717227 |
| 3 | 55.785672821 | 0.2654892935 | 0.1042404541 |
| 4 | 55.741234408 | 0.1589794007 | 0.0385846629 |
| 5 | 55.725272951 | 0.0959816052 | 0.0141978278 |
| 6 | 55.719451564 | 0.0581176321 | 0.0052233987 |
| 7 | 55.717316746 | 0.0352283035 | 0.0019216203 |
| 8 | 55.7165323 | 0.0213621834 | 0.0007069298 |
| 9 | 55.716243841 | 0.0129557347 | 0.0002600656 |
| 10 | 55.71613774 | 0.0078578084 | 0.0000956729 |
| 11 | 55.716098709 | 0.0047659477 | 0.0000351961 |
| 12 | 55.716084351 | 0.0028906814 | 0.0000129479 |
| 13 | 55.716079069 | 0.0017532842 | 4.7632756e-6 |
| 14 | 55.716077126 | 0.00106342 | 1.7523112e-6 |
| 15 | 55.716076411 | 0.0006449967 | 6.4463927e-7 |

## Whole Model Test

| Model | -LogLikelihood | DF | ChiSquare | Prob>ChiSq |
|---|---|---|---|---|
| Difference | 13.518620 | 4 | 27.03724 | <.0001* |
| Full | 55.716076 | | | |
| Reduced | 69.234697 | | | |

| | |
|---|---|
| RSquare (U) | 0.1953 |
| AICc | 122.07 |
| BIC | 134.458 |
| Observations (or Sum Wgts) | 100 |

## Fit Details

| Measure | Training | Definition |
|---|---|---|
| Entropy RSquare | 0.1953 | 1-Loglike(model)/Loglike(0) |
| Generalized RSquare | 0.3160 | (1-(L(0)/L(model))^(2/n))/(1-L(0)^(2/n)) |
| Mean -Log p | 0.5572 | ∑ -Log(ρ[j])/n |
| RASE | 0.4343 | √ ∑(y[j]-ρ[j])$^2$/n |
| Mean Abs Dev | 0.3782 | ∑ \|y[j]-ρ[j]\|/n |
| Misclassification Rate | 0.2600 | ∑ (ρ[j]≠ρMax)/n |
| N | 100 | n |

## Lack Of Fit

| Source | DF | -LogLikelihood | ChiSquare |
|---|---|---|---|
| Lack Of Fit | 7 | 2.168737 | 4.337474 |
| Saturated | 11 | 53.547340 | Prob>ChiSq |
| Fitted | 4 | 55.716076 | 0.7402 |

## Parameter Estimates

| Term | | Estimate | Std Error | ChiSquare | Prob>ChiSq |
|---|---|---|---|---|---|
| Intercept | Unstable | 8.15677855 | 775.19788 | 0.00 | 0.9916 |
| major adverse cardiac event[not diseased] | | -0.531345 | 0.2612072 | 4.14 | 0.0419* |
| acute aneamia due to bleeding[not diseased] | | -0.4248407 | 0.2358903 | 3.24 | 0.0717 |
| sepsis, all categories[not diseased] | | -0.7406676 | 0.2410937 | 9.44 | 0.0021* |
| thrombosis or embolism[not diseased] | Unstable | -7.8811169 | 775.19789 | 0.00 | 0.9919 |

For log odds of dead/survived

## Effect Likelihood Ratio Tests

| Source | Nparm | DF | L-R ChiSquare | Prob>ChiSq |
|---|---|---|---|---|
| major adverse cardiac event | 1 | 1 | 4.36773882 | 0.0366* |
| acute aneamia due to bleeding | 1 | 1 | 3.24971141 | 0.0714 |
| sepsis, all categories | 1 | 1 | 9.99453598 | 0.0016* |
| thrombosis or embolism | 1 | 1 | 3.8601677 | 0.0494* |
